# Risk factors for school-based presenteeism in children: a systematic review

**DOI:** 10.1186/s40359-023-01207-1

**Published:** 2023-05-23

**Authors:** Lisa Woodland, Samantha K. Brooks, Rebecca K. Webster, Richard Amlôt, G. James Rubin

**Affiliations:** 1 Department of Psychological Medicine, King’s College London, London, UK; 2 Department of Psychology, University of Sheffield, Sheffield, UK; 3 Behavioural Science and Insights Unit, Health Security Agency, Salisbury, United Kingdom; 4 NIHR Health Protection Research Unit in Emergency Preparedness and Response at King’s College London, London, United Kingdom

**Keywords:** Presenteeism, School, Children, Systematic review, Illness, Infectious disease, Parents

## Abstract

**Introduction:**

Children attending school whilst unwell, known as school-based presenteeism, results in negative impacts on education and mental and physical health. We aimed to identify the risk factors for this behaviour.

**Method:**

We conducted a systematic search of five databases (11 July 2022) using words associated with school (e.g., school and childcare) and presenteeism (e.g., presenteeism and sick leave). The studies are synthesised according to the risk factors associated with school-based presenteeism and are grouped into themes by related topics.

**Results:**

Our review included 18 studies, with quantitative, qualitative, and mixed-method study designs. Children, parents, and school staff reported past incidents and intentions for future presenteeism. We identified five themes from these reports: perceptions about the illness / signs and symptom(s); children’s characteristics; children’s and parents’ motivations and attitudes towards school; organisational factors; and school sickness policy. Increased risk of school-based presenteeism was commonly linked to symptoms that were perceived low in severity and unidentifiable, children with a high school absence record, disbelief in children’s illness, unsupportive employers, vague school policies and financial consequences.

**Conclusions:**

School-based presenteeism is complex due to the competing interests of the multiple individuals involved, such as children, parents, and school staff. Sickness policies need to include clear and specific guidance about illness and the signs and symptoms of diseases and should be communicated to all relevant individuals to mitigate against discrepancies in how the policy is interpreted. Furthermore, parents and school staff need support, such as financial and childcare, to be able to manage children when they are unwell.

**Supplementary Information:**

The online version contains supplementary material available at 10.1186/s40359-023-01207-1.

## Introduction

Presenteeism commonly describes someone who is present at work despite being unwell, and has been described as the counterpart to absenteeism [1]. Although one of the first studies to review presenteeism identified eight different definitions [2], and a recent review suggests that there is still little consensus on how presenteeism is defined and measured [3]. In this paper we are interested in presenteeism among school children, which is also often not specified within the literature. Therefore, we have identified school-based presenteeism and defined it as ‘a child attending school for any period, whilst unwell.’ Workplace presenteeism has more societal costs than absenteeism [4] and is estimated to cost £15.1 billion annually in the UK [5]. Presenteeism has also been shown to increase the risk of long-term adverse physical (e.g., diabetes, arthritis, back pain and headaches) and mental health (e.g., depression, bipolar and anxiety) problems [6]. Research about the effects of school-based presenteeism on children’s health and education is limited.

In the UK, the COVID-19 pandemic brought presenteeism into the foreground as attending school or work whilst experiencing a cough, fever or loss of (or a change in) taste or smell was strongly discouraged [7]. Other infectious diseases, such as influenza and gastrointestinal diseases, are prevalent within the UK population [8, 9], and requests for people to stay at home whilst they are unwell is not new [10]. Despite this, 88% of people working in UK colleges and universities reported working whilst sick “some” of the time [11], while 70% of UK parents have admitted to sending their children to school or nursery “often” or “occasionally” when they were ill, and 17% of children had been sent to school with vomiting, 18% with diarrhoea and 25% with a high temperature [12]. This is in contravention of official guidance [13].

There is a growing volume of research about factors associated with presenteeism in the workplace [2, 3]. Factors associated with presenteeism in children have been less well explored. Outbreaks of infectious illnesses within educational settings are common, particularly in England’s primary schools [14], which can lead to increased rates of hospital attendance among children [15], impacting children’s health and education. One study in Peru, among university students also found a significant association between presenteeism and reduced academic performance, which had a greater effect size than the impact of absenteeism on academic performance [16]. For students who reported presenteeism, most found it difficult to concentrate in class (96%) and reported being tired (87%), distracted (82%) and studying slower (77%).

Several factors may play a role in exacerbating school-based presenteeism. Under the law, children in the UK cannot be left alone if it places them at risk [17]. Therefore, employed parents may need to take time off work to supervise their children when they do not attend school. As such, the risk factors for presenteeism connected to employment may also be relevant, via parental behaviour, to school-based presenteeism. Previous research suggests that these risk factors are: type of occupation; worries and or concerns about employment (e.g., lack of work cover, increased colleagues’ workload, and might miss vital information), pay and job loss as a result of not attending work; and social norms within the organisation [18–20]. Non-employment risk factors may also be relevant, including factors relating to attitudes towards schooling and towards infectious illness, and policies within schools.

In this study, we conducted a systematic review to identify the risk factors associated with children attending school despite being unwell. Throughout, we are neutral as to whether it is or is not appropriate for children to attend school with any given set of symptoms. Instead, we address the narrower question of what affects adherence to such policies.

## Method

The protocol is registered on PROSPERO (ID CRD42020167344).

We reported data using Preferred Reporting Items for Systematic reviews and Meta-Analyses [21].

### Search strategy

An initial scoping review was conducted whereby LW entered “presenteeism” into Medline and searched PROSPERO. We took this first step to review the literature about presenteeism, to prevent conducting a study that was already present within the literature and to guide the design of our search strategy. As well as using previous literature, the search terms were also guided by a search strategy that we had previously conducted [19]. LW and GJR tested various search strategies to balance the number of search results and the relevance of article topics. During this phase we also assessed the accuracy of our search strategy; if studies were not present that we had identified from our scoping review as potentially relevant, we modified our search strategy. LW, RKW, RA and GJR developed the search strategy. We used terms and words associated with school (e.g., school, childcare, and nursery) and presenteeism (e.g., presenteeism, sick leave, and unauthorised absence). We used the Boolean operators AND, OR and wildcards (e.g., *) to expand or narrow the search. The search strategy was modified to meet the requirements of each database. The search strategies for each database are provided as Additional files 1.

### Searches

We searched: Medline (1946 to 20 January 2020), APA PsycInfo (1806 to 21 January 2020), Child Development and adolescence development (all years to 22 January 2020), APA PsycArticles (1894 to 24 January 2020), and Web of Science (1956 to 24 January 2020). These databases were chosen to cover social and health sciences and children [22]. Because of an unintentional extended period between when the initial search was conducted and the analysis, we repeated these searches on 11 July 2022 for articles published in 2020 and onwards so that the search was up to date.

### Review process

LW combined the electronic searches from each database into Endnote [23], and removed the duplicates. The titles and abstracts were screened for mentions of presenteeism within schools. A full-text review was conducted if the content of the study was not clear from the abstract. Potentially relevant studies were then screened against the inclusion criteria. The reference lists of articles that met our inclusion criteria were searched for any additional potential studies.

### Selection criteria

Studies that met the criteria outlined below were eligible for inclusion in the review:

#### Population

Children under 19 years old enrolled in school. “School” includes pre-schools (e.g., nurseries and other types of day-cares).

#### Exposure

Data reporting the risk factors associated with presenteeism by children, parents or school staff.

#### Outcome

Intentions and actual presenteeism behaviour in relation to school. There were no specific requirements with how presenteeism was measured, although the outcome needed to meet our definition of “school-based presenteeism,” which was defined as a child attending school for any period whilst unwell, and our definition of “unwell” included chronic and acute illness [1]. For example, a child who reports that in the past 12 months they had attended school when they should have stayed at home because they were unwell or a parent who reports that they had sent their child to school with a temperature.

#### Comparators

Studies were excluded that considered minor chronic illness (e.g., hay fever) and where schools actively promoted school attendance for children with a given chronic illness. These studies were excluded for clarity; it was apparent that these children, although were unwell were expected to attend school. But if the study reported otherwise, the study was included.

#### Study design

There were no limitations on the study design. Articles that did not report on original data were excluded (e.g., commentaries and editorials).

#### Other limiters

Only studies published in English were included as this is the language spoken by the reviewers.

### Data extraction

LW extracted data from the included studies using a data extraction table designed for this systematic review. Data were also extracted from a subset (50%) of the included papers by a second author (SKB) (Cohen’s Kappa percent agreement of 89% [24]). The data extracted included: citation, country of study, study design, sample characteristics (age and gender of participants and children), type of school, illness, and risk factors associated with presenteeism (see Additional files 2).

### Quality assessment

LW assessed each study using the Mixed Methods Appraisal Tool (MMAT) [25]. Each study was examined against five criteria for each type of study design included in the paper and scored “yes,” “no,” or “cannot tell” depending on whether the study met the criteria. For example, qualitative studies were assessed against the following criteria: (1) whether their approach was appropriate to answer the research question; (2) whether the data collection methods were adequate to address the research question; (3) whether the findings were adequately derived from the data; (4) whether the interpretation of the results was sufficiently substantiated by data; (5) whether there was coherence between qualitative data sources, collection, analysis and interpretation. SKB quality assessed 50% of the included papers - only a sub-sample was double-assessed due to consensus in assessment between authors.

### Data synthesis and analysis

We chose this method because of the expected heterogeneity in study designs and outcomes. The studies are synthesised according to the factors associated with presenteeism and are grouped into themes by related topics. The effect measure(s) that relate to our study aims will be described for each study, such as odds ratio (OR) and frequencies (%) for quantitative results and a description of the study themes for qualitative results.

## Results

### Search results

**Fig. 1:**
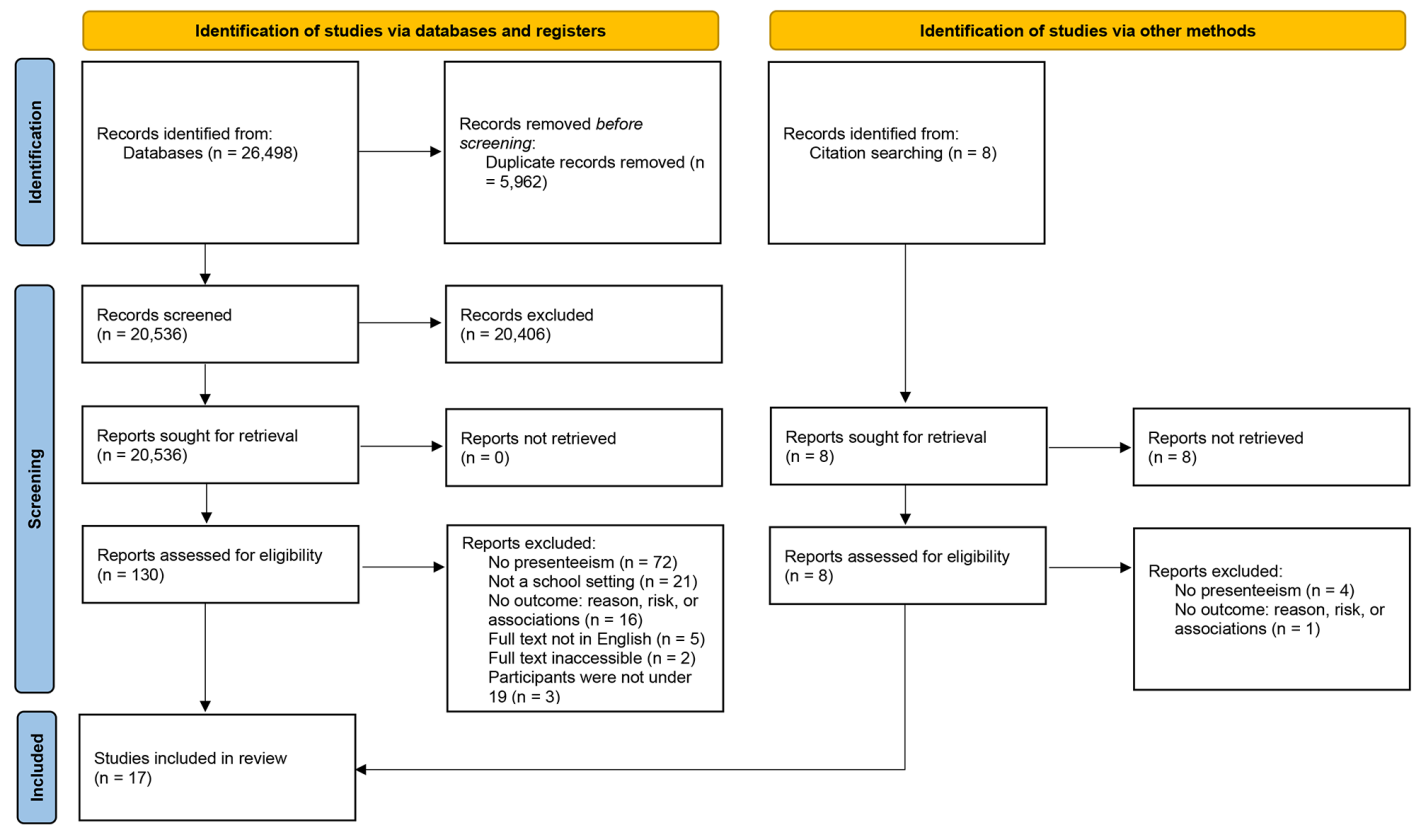
PRISMA flow diagram for search one of two conducted in 2020 displaying the screening process and reason for study exclusion

Figure 1 displays the 2020 search that produced 26,498 records from the databases and eight from searching reference lists. After screening, 17 studies were eligible for inclusion.

**Fig. 2:**
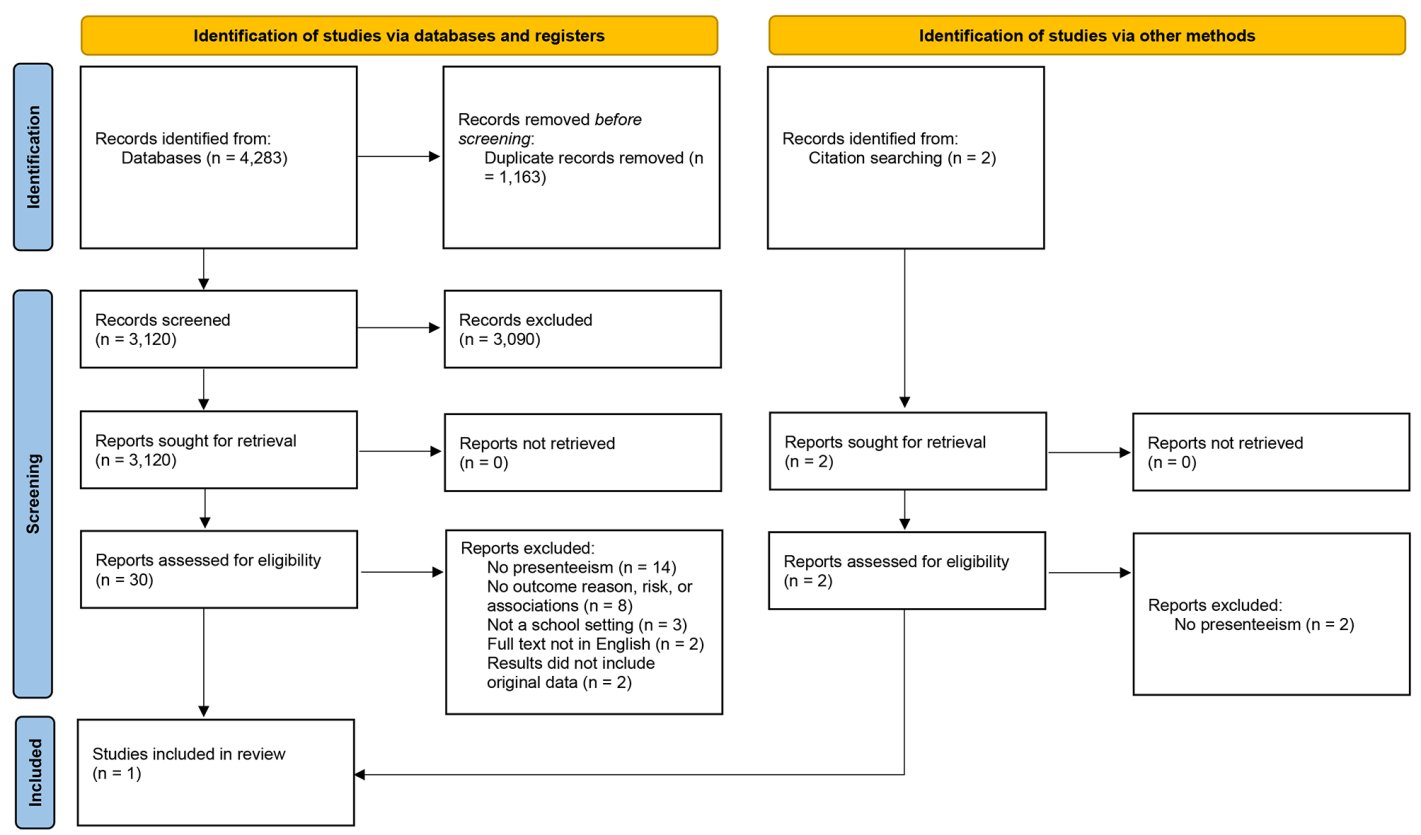
PRISM flow diagram for search two of two conducted in 2022 displays the screening process and the reasons for study exclusion

Figure 2 displays the 2022 search that produced 4,283 records from the databases and two from searching reference lists. After screening, 18 studies were included in the review.

### Study characteristics

Most studies were conducted in the UK (39%, n = 7) [26–31] followed by US (28%, n = 5) [32–36] and Canada (11%, n = 2) [37, 38]. Three countries had one study only: Australia [39], Norway [40], and Switzerland [41]. Two studies (11%) had participants drawn from several countries (Belgium, Estonia, Finland, Italy, and Latvia) [42, 43].

A variety of study designs were used, including quantitative (61%, n = 11) (cross-sectional survey [32–36, 38, 40, 41, 43], discrete choice experiment [26], repeated measures [42]); qualitative (22%, n = 4) (interviews [27, 39], ethnography [28, 29]); and mixed-methods (17%, n = 3) [30, 31, 37].

Parents and school staff were the participants in most studies (39%, n = 7) [30–35, 37], followed by studies that investigated parents (28%, n = 5) [26, 27, 29, 36, 39], children (17%, n = 3) [40, 42, 43] and school staff (17%, n = 3) [28, 38, 41] individually.

Presenteeism was reported in five different school settings: formal pre-school (for children five years and under) (56%, n = 10) [26, 27, 30, 32–35, 38, 39, 41]; formal and informal pre-school (11%, n = 2) [31, 37]; primary school (children aged five to 11 years) (11%, n = 2) [28, 29]; secondary school (children aged 11 to 19 years) (17%, n = 3) [40, 42, 43]; and a non-specified school setting in which children were aged between eight and 15 years (5%, n = 1) [36].

Half of the included studies reported presenteeism in children with a non-specified illness, such as children who were “marginally unwell,” “too sick for school” and “ill” (50%, n = 9) [26, 28, 29, 37, 39–43]. The other studies reported presenteeism in children with specific diseases or symptoms (50%, n = 9), which were: respiratory tract infections (RTIs) [27, 34, 38]; gastrointestinal infection (Salmonella) [30]; infectious illnesses [31]; stomach ache or abdominal pain [36] and several signs and symptoms of illness [32, 33, 35], such as one study which reported eight different symptoms (a new runny nose, new cough, unusually cranky, ear pain, sore throat, skin rash, diarrhoea and conjunctivitis) and four different temperature ranges. The narrative results report the terminology that is used in the included studies, although “unwell” has been used throughout when the study describes a non-descript illness or signs and symptoms of illness for clarity.

### Quality assessment

Of the eleven quantitative studies, the overall study quality was low: only four studies recruited participants from more than one location [36, 40, 42, 43]; few studies used a standard validated measure [36] or validated the internal consistency of the measures used in the study [34, 43]. Other methodological criteria were often not reported: it was unclear which variables were used in one study [38]; how missing data were managed in three studies [36, 40, 42]; and how the variables were analysed in two [38, 41].

All four qualitative studies had an approach appropriate to answer the research question, but the data collection and analysis were often described inadequately, which reduced the overall quality of the studies. No studies used a recruitment strategy adequate to address the research question, and two studies reported on a select group of participants from one location (e.g., a city) [27, 39]. In the two ethnographic studies, the reason for choosing the case subjects was not described [28, 29]. All studies used quotes to support the themes identified, although the process that was used to interpret the data into themes was poor. However, one study reported that a second author reviewed a sub-section of the findings to validate the themes that were identified [27].

Of the three studies using mixed-methods, one was of low quality [37] and the other two high [30, 31]. The study of low quality did not clearly justify the reasons for using a mixed-method; describe how the data had been collected; describe the analysis process; include participant characteristics; or integrate the findings from the qualitative and quantitative data [37]. One high quality study met the quality assessment in the five criteria in the qualitative and quantitative components of the study [31]. The second high quality study met all the criteria, except quotes were not used to support the qualitative findings.

#### Risk factors associated with school presenteeism

The main effects and characteristics of the included studies are shown in Additional files 2. Studies reported the risk factors associated with presenteeism by reporting previous experience of and intentions regarding presenteeism. In the five studies that reported presenteeism prevalence: 69% of children reported at least one episode of presenteeism [30, 40, 42] (50%, 77.5%, 79.5%, respectively), 48% of children reported two or more episodes of presenteeism [43], and 43% of parents reported they would send their marginally unwell child to school [26]. There were five themes in the results: perceptions about the illness / signs and symptom(s); children’s characteristics; children’s and parents’ motivations and attitudes towards school; organisational factors (including the school and parents’ employers); and the school sickness policy. The five themes, illustrated with aspects that heighten or reduce the risk of presenteeism are presented in Table 1.

**Table 1:** Five themes that impact school-based presenteeism and the risk and mitigation factors linked to the theme

Themes that impact school-based presenteeism		
Factors that increase risk of presenteeism		Factors that decrease risk of presenteeism
- Conflicting symptom perceptions between relevant individuals* - Symptoms attributed to alternative causes	**Perceptions about the illness / signs and symptom(s)**	- Identifiable and measurable (e.g., a temperature) - Severe or contagious
- High school absence	**Children’s characteristics**	- Country of education - Relevant individuals* believe children’s claims of illness
- Children with high motivations (e.g., interest and enjoyment) toward school - Children that were worried about lost education - Children in transition periods	**Children’s and parents’ motivations and attitudes towards school**	- Parents that perceive presenteeism as unacceptable
- Lack of childcare - Parents had employment worries - School staff that feel pressured to keep unwell children in school - Lack of medical knowledge among relevant individuals*	**Organisational factors (including the school and parents’ employers)**	- Parents’ employers support them when children were unwell - Parents perceive school staff manage unwell children appropriately - Parents were concerned about their unwell children - Policies that penalise schools for ineffectively managing unwell children
- Policies that are vague about inclusion and exclusion criteria - Policies that accept children who are taking medication for the illness (prescription and non-prescription)	**School sickness policy**	- Policies that mitigate the financial consequences associated with children staying at home when unwell - Policies that adequately reflect day-to-day practices

* Relevant individuals include parents, children, or school staff

#### Perceptions about the illness / signs and symptom(s)

Four studies reported that children with a high temperature were at lower risk of presenteeism compared to children with other symptoms of illness [32, 33, 35, 41]. Participants more often reported symptoms that related to temperature (e.g., “mild febrile illness” and “fever”) as a reason to exclude children from school compared to the other symptoms listed [33, 41]. Specifically, nearly all parents (94%) believed that school staff ought to exclude a child with a temperature above 101℉ (38.3℃) and 99% of school staff indicated they would exclude a child with this temperature [32] or 102℉ (38.89℃) (parents = 93% and school staff = 97%) [35]. When studies compared exclusion rates using different illness scenarios, the intended rate of exclusion increased when a high temperature was included in the scenario for parents and school staff [33, 35].

Five studies suggested that children with diarrhoea were at lower risk of presenteeism compared to children with RTIs and RTI-like-symptoms (excluding a high temperature) [27, 32–34, 38]. One study found that parents and school staff were more adherent to sickness guidelines about diarrhoea compared to RTIs [33]. Parents appeared to be of the opinion that they would not send children to school with diarrhoea but were less certain about what to do when children had coughs and colds [27]. In one study parents and school staff reported higher rates of school exclusion for children with “more than three loose stools” (diarrhoea) compared to “wheezing” and “uncontrolled coughing” [32]. Regarding RTIs but not diarrhoea, fewer than 35% of parents and school staff reported that children with an RTI and one of three additional symptoms (clear runny nose, green runny nose, and cough without difficulty breathing) should be excluded from school [34]. However, one study found that over half of school staff would exclude a child when they had an RTI and ear pain (64%) or green or yellow nasal discharge (56%) [38].

Three studies reported about conjunctivitis and in each study, there were instances (e.g., reported by parents or school) where conjunctivitis was reported more frequently as a reason to exclude children from school than diarrhoea [33, 35, 41]. Two studies reported about vomiting, and in both studies, vomiting was reported more often than diarrhoea, as a reason for exclusion [32, 41]. Two studies identified that parents and school staff frequently reported signs about children being less active and requiring more care than usual as a reason to exclude them from school [32, 38]. Children who were “persistently crying” [32], displayed “unusual behaviour,” “cough with phlegm” [38], “skin rash” and “ear pain” [35] were less frequently reported as a reason to exclude children from school compared to other symptoms.

Perceptions about illness contagiousness and severity appeared to impact the risk of presenteeism. Six studies linked presenteeism with whether the illness was perceived as “contagious” [27, 31, 37–39, 41]. One study suggested that although parents reported they would not send their children to school whilst they were contagious, parents also described intentions about presenteeism that contradicted this statement and did not seem to understand the meaning of contagious [27]. Similarly, school staff in one study reported that they would exclude children when they had an illness that they perceived to be contagious, but they were unsure when the illness was contagious and suggested non-infectious causes for symptoms (e.g., teething) [41]. There appeared to be little consistency between participants as to the signs and symptoms that indicated a contagious illness, although when the symptoms were perceived as contagious the risk of presenteeism reduced [31, 37–39, 41].

Five studies suggested that the risk of presenteeism was reduced when the symptoms were perceived as “severe” [27, 28, 30, 33, 41]. One qualitative study suggested that there were “grey areas,” and parents reported sending their children to school when they appeared to be unwell because the symptoms were not severe enough; parents perceived the illness was severe when the symptoms impacted children’s temperament / general demeanour and as low severity when there was only one (unspecific) symptom of illness [27]. Another qualitative study reported that school staff would only consider sending unwell children home from school when they had sat quietly for between 15 and 30 min and were still reporting they were unwell or when the symptoms were “dramatic, threatening and visible” [28].

#### Children’s characteristics

Five studies explored the association between children’s characteristics and presenteeism [28, 29, 36, 40, 42]. Their findings were mixed. In two studies that investigated the same characteristics, a higher risk of presenteeism was found in one of the studies for children that were: girls compared to boys [40]; immigrants compared to natives [42]; and taking a vocational course (a course that leads to a craft) compared to general studies [40]. However, the alternative study found no significant differences between gender [42], residency status [40] and course type [42]. But both studies found that children with high levels of school absences were at higher risk for presenteeism than children with low school absences [40, 42]. In addition, one of the studies found children from Latvia, Estonia, and Italy were at higher risk (in order of highest to lowest increase in risk) compared to children from Finland [42].

One study suggested that children’s characteristics had low importance and reported that most parents and school staff believed school staff had good judgment and were consistent about which children needed to be excluded and which did not [32]. One qualitative study identified that some children were more likely to be believed about their illness by school staff compared to other children [28]. A second publication reporting about the same children, although describing the mothers’ experiences, suggested that parents not believing their children’s claims of illness was also a risk factor for presenteeism [29]. The study suggests that when children claim they are unwell, mothers first consider whether the claim is “real” or “feigned”, and if considered real, the mother then decides whether the claim is due to them being unwell or an emotional problem or upset; only when the parent accepts that the symptoms are “real” and due to illness will action be taken such as treatment and keeping the child home from school [29]. One study found that children with no siblings were less likely to miss school compared to children with siblings when they have a stomach ache or abdominal pain [36]. The study suggested that parents with one child often discount, criticise, or ignore their child’s pain complaints [36]. Maternal responses to children’s illness behaviours differed between the two groups, although there were no statistically significant differences.

#### Children’s and parents’ motivations and attitudes towards school

Four studies suggested that children’s motivation toward school affects the risk of presenteeism [29, 40, 42, 43]. Children were at higher risk of presenteeism when they had high motivations about school (e.g., when they were interested in school and liked schoolwork) [40, 42, 43] and worried that they might miss important information if they did not attend school [43]. Parents of children in their last year of primary school reported themes that encouraged presenteeism, one of which about “emotional upset and training in stoicism” suggested that children need to learn to “cope” with illness because it will be difficult to “get away with” feigning illness at secondary school [29]. In addition, children that were in their final year of school were more likely to engage in presenteeism compared to children in the previous school year [42]. Similarly, children in their last years of school commonly reported the reason for presenteeism was that absence from school would impact their career prospects [43].

One study suggested that parents had an “unwritten rule” that presenteeism was unacceptable, and parents were frustrated when they suspected their children had caught an illness at school because other parents had not abided by the rule [27]. However, in the same study, parents also acknowledged that parents generally tried to make the best decisions, and that other parents who had sent children to school whilst unwell had the same pressures and dilemmas they had had [27].

#### Organisational factors (including the school and parents’ employers)

Three studies suggested that reasons for presenteeism included parents being unable to take time off work and find alternative childcare [30, 38, 41]. A fourth study suggested that presenteeism would increase if the school had a quiet room for unwell children [26]. In connection, there was a higher risk of presenteeism when employed parents felt a responsibility to go to work, and were concerned about the burden and increased workload of colleges and that colleagues would perceive them negatively if they took time off work to care for children who were too unwell for school [27]. One study found parents and school staff frequently reported that employers supported parents’ need to care for their unwell child and only 17% of parents felt that how school staff handled unwell children negatively affected their job success [32]. One study reported that parents would not be able to concentrate at work because they would be too worried about their unwell children if they sent them to school and therefore, they would take time off work to care for them at home [37].

Three studies about school staff reported concerns about having unwell children in school, which facilitated their decision to exclude unwell children. School staff reported that unwell children increased the staff’s workload, and they did not have the space and resources to care for unwell children [37, 38, 41]. School staff were also concerned about the school’s liability and a lack of legislation and funding if they cared for unwell children at school [37]. However, two of the studies reported that staff had kept unwell children in school because of pressure from parents [37, 38]. One study suggested that presenteeism occurred because parents did not communicate when children had symptoms of illness when they dropped them off at school [41]. The same study also identified that a lack of medical knowledge and conflicting information from medical sources was a barrier to school staff making an informed decision about exclusion [41]. One study found that parents were influenced by school staff’s recommendations on how to manage children’s illnesses [31].

#### School sickness policy

One study found that 18% of formal pre-schools and 41% of informal pre-schools did not mention specific infections and criteria for exclusion and readmittance in their sickness policies [31]. The study also reported that parents and school staff believed the sickness policies were an accurate reflection of their day-to-day practices [31]. One study found that parents suggested the sickness policies were vague, particularly for RTI symptoms compared to gastrointestinal illnesses and the clear timescales for how long children need to be excluded from school with illnesses resulted in presenteeism [27]. In another study, parents (31%) and school staff (51%) did not perceive that sickness policies were too vague and that school staff followed the written exclusion guidelines closely (parents = 78% and school staff = 86%) [32].

One study found that more than double the amount of parents would send unwell children to school if the sickness policy allowed children to take paracetamol (paracetamol allowed = 62% and paracetamol not allowed = 25%) [26], and parents also believed that they could send a child back to school whilst unwell if they had taken antibiotics [31]. Two studies found that school staff would also keep children at school if the child had a prescription (antibiotics) for the child’s illness [38] and used drugs that reduced a high temperature [41].

Four studies identified factors relating to the financial consequences of not sending a child to school, such as lost fees (e.g., lack of reimbursement for paying pre-school fees upfront), lost wages [27, 37] and fear of job loss after taking time off work to care for unwell children [37]. Intentions about presenteeism were reduced if parents had the option to swap unused pre-school sessions or receive reimbursement for unused sessions [26].

## Discussion

School-based presenteeism, whereby children attend school despite being unwell, is a complex process, with decisions involving the children but also, primarily, their parents and school staff. The findings from our review suggest three stages in the presenteeism decision process: (1) parents must decide whether the child is unwell (and acknowledge illness); (2) factors external to the illness are considered (children’s characteristics, attitudes and motivations, organisational factors, and school sickness policy); (3) a decision about whether the child attends school / is sent home from school is made.

When children are unwell, whether at home or school, the illness needs to be acknowledged before a decision about school attendance can be made. The symptoms present appear to impact whether relevant individuals (e.g., children, parents, or school staff) acknowledge the illness. Without illness acknowledgement, the risk of presenteeism is increased. Children with temperatures, or symptoms that are perceived as severe and contagious, were consistently more likely to be kept out of school, either because parents do not send them to school or because school staff exclude them from school. In contrast, symptoms that are considered less severe or not contagious result in less clear action. This finding aligns with previous research about workplace presenteeism which suggests symptoms perceived as mild increase the risk of presenteeism [18, 19]. The link between concern about a child’s illness when a temperature is present is unsurprising. Research routinely suggests that parents are concerned about a high temperature [44–47]. While this is largely appropriate, a runny nose, nasal congestion, and cough, which were considered less severe, are symptoms commonly caused by RTIs [48, 49], and have previously been used as indicators that a child should remain at home, particularly during the COVID-19 pandemic. In addition, research identified in our review has highlighted that parents can have a poor understanding of “contagious.” It is important to be clear with parents and schools about what signs and symptoms of illness children can and cannot attend school with.

Child characteristics also influence both the recognition of illness and the subsequent decision-making process. One of the highest motivations for presenteeism was that high absence might negatively affect grades, while children who already had high levels of absence from school were at higher risk of presenteeism than those with low school absences. There is a difficult balance to make here. Poor school attendance affects children’s educational attainment, social development, employment, and mental and physical health outcomes [50–52]. As such, programmes and procedures are in place to encourage school attendance. For instance, some schools promote school attendance by rewarding individual children and school classes for good attendance and fining parents for child absences [53]. On the other hand, although the research about presenteeism among children is limited, there is evidence to suggest that presenteeism may adversely impact children’s health and education [15, 16] as well as contributing to the spread of infection. More research is needed to explore the full extent of the health and educational effects of presenteeism among children. However, to reduce presenteeism, schools may need to send clearer messages that stipulate that school absence due to illness is appropriate and expected.

The relationship between gender and presenteeism was inconclusive. The mixed findings about gender that we identified mirror the findings about gender and workplace presenteeism [2, 18, 19, 54]. When significant gender differences were found studies commonly report female employees to be at higher risk of presenteeism compared to male employees [18, 19], which aligns with our findings. Of the two studies that reported about gender, one study found females were at higher risk of presenteeism, the other showed no significant differences. A previous study about university students also indicated that presenteeism occurred more frequently in female students [16]. Thus, it could be argued that females are at higher risk of presenteeism, although we suggest nuances in the *role* of gender may explain the apparent differences between studies. For example, motivations for presenteeism may differ by gender, with girls more motivated by extrinsic factors (e.g., attendance pressure and to get good grades) whereas boys are motivated by intrinsic reasons (e.g., well-being at school) [43]. School staff also appeared to believe girls less often than boys when students claimed they were unwell, while parents appeared to perceive sickness in boys and girls differently [28, 29]. Similar findings have been found in a study about children with chronic illnesses [55]. In a previous UK survey, the number of reported presenteeism episodes appeared to differ between parent gender [12]. When parents were asked to report how often they had sent their children to school whilst ill, “often” was reported by 13% of female parents compared to 31% of male parents. Similarly, “often” was reported by 14% of female parents compared to 38% of male parents when asked about sending their children to school with a contagious infection. This needs further investigation; we are unsure if these findings reflect a difference in perception of illness, attitudes towards school attendance, or how mothers and fathers respond to questionnaires.

With respect to children’s and parents’ attitudes and motivations, we found that children who had more interest in school were at increased risk of presenteeism, which mirrors findings about workplace presenteeism [18, 19]. We also found that motivations and risk of presenteeism appeared to alter depending on school year. The risk of presenteeism increased during important school years, such as those with exams and in transitional school years, something that may help explain why, during the COVID-19 pandemic in England, children in important “transition years” were more likely to attend school soon after experiencing COVID-19 like symptoms compared to other children [56].

In terms of organisational factors, a prominent risk factor for presenteeism was the lack of availability of alternative childcare when children were unwell. A previous study found that a main reason for parents to disagree with an unexpected school closure related to difficulties in finding alternative childcare and the knock-on financial impacts if parents needed to take time off work [57]. In studies in the current review, organisations that supported parents to take time off work appeared to reduce the risk of presenteeism. These findings align with the findings from research about workplace presenteeism [18, 19].

By law, schools in the UK have to provide a space to treat sick or injured pupils [58] and safeguard children’s mental and physical development [59]. School staff’s concerns about being unable to care for unwell children adequately was a reason to send them home when unwell. Similarly, one study suggested that irrespective of organisational pressures, parental worry about their unwell children would prevent them from attending work as they would want to care for their child, in line with research on full-time working mothers that found that “being there” for their children was a primary concern [60]. These views emphasise that parents and school staff have the same goals, to protect the health and well-being of children. But we observed a barrier on both sides; school staff commonly perceived parents were dishonest when children were unwell, and parents felt unable to be honest, although they usually were. This barrier has been identified before, particularly in the connection between using medicines to speed up children’s illnesses and to mask symptoms of illness [34, 61–63]. Promoting that schools are responsible, aim to maintain children’s good health and that they understand parents may have organisational pressures upon them, may enhance the dialogue between parents and school staff. As a result, discussions about school attendance will be more informed and appropriate decisions about school attendance may increase.

School sickness polices were also found to affect presenteeism. Vague policies seem to be particularly unhelpful. In the UK, the Government provides guidance about when children should not attend school because of illness, which includes specific childhood diseases such as chicken pox and symptoms such as diarrhoea and vomiting [13]. However, there are still vague sections. For example, children with influenza are recommended to stay out of school “until recovered.” Schools are also at liberty to create their own guidance. Implementation of sickness policies can therefore leave scope for misinterpretation or misapplication. Parents and school staff commonly agreed on which signs and symptoms of illness children should not attend school with. However, parents and school staff showed higher rates of adherence to decisions about school attendance for some illnesses compared to others. The findings suggest that adherence increases when parents and school staff are *sure* about what action to take. This mirrors previous research that suggests that uncertainty and confusion about health information increase the risk of non-adherence to health behaviours [64–66]. More explicit guidelines are likely to increase adherence to sickness policies.

The importance of not having in-person social contact and attending work or school when presenting with symptoms of an infectious disease was heightened during COVID-19. As such, consideration needs to be given to the lack of studies that were conducted during and or after the pandemic and the impact of this on the review’s findings. Still, school outbreaks of COVID-19 were common, and evidence suggests that children attended school when they had symptoms of COVID-19 [56]. Research about the risk factors associated with presenteeism during the pandemic is emerging. However, the research is about workplace presenteeism [67–70]. School-based presenteeism needs specific investigation and as a priority because of the already limited research in this area. This study shows that school-based presenteeism is a unique issue and that the risk factors associated with children attending school whilst unwell differ from that of workplace presenteeism. Moreover, as well as the educational impacts, the health impacts are also likely to be distinct from workplace presenteeism and, therefore, must be explored.

### Quality of included studies

The majority of studies included in this review were of low quality due to studies’ sampling methodology, targeted populations, and insufficient analysis descriptions. These omissions suggest that the study findings are specific to the study population rather than broader populations. Moreover, 50% of the studies were from two countries, UK and US, which compounds this limitation. However, these limitations have a minimal bearing on the reliability of the results. In the quantitative studies, most had appropriately measured and reported the outcome, accounted for confounders, and used statistical analysis appropriate to the research question. In qualitative studies, the interpretation of the results was sufficient and supported by the data.

### Quality of this review

This review highlights that there are many gaps in the literature about presenteeism. First, most of the findings were primarily self-reports from female parents; a small number of responses from male parents and children were included in the review. Second, our review outcomes may have been impacted if we had discussed our findings by children’s age rather than by studies’ response type (parents, children or school staff). Third, previous research about workplace presenteeism suggested other factors not identified in the school literature, such as self-perceptions about health and control over life, may impact the risk of presenteeism. Fourth, the variety of illnesses that are explored is limited; reviewing different illnesses could affect the review findings. Fifth, perceptions about symptoms, illnesses and policies may have changed due to the COVID-19 pandemic; therefore, studies that investigate presenteeism post-pandemic are needed to corroborate the review’s findings. Sixth, studies of interventions to change presenteeism are entirely absent in the literature. Finally, how the included studies measured presenteeism varied; our definition described being in school whilst unwell for “any period,” which may have affected our findings. Future research needs to investigate a standard measure and definition of school-based presenteeism so that research about presenteeism, including potential interventions are reliable.

## Conclusion

Eighteen studies were analysed to identify the risks concerning school-based presenteeism. These studies suggest that presenteeism stems in part from a failure by parents and school staff to identify and acknowledge children’s illnesses and to accept children’s claims of illness. Other factors such as children’s characteristics, motivations and attitudes towards school, organisational factors (including the school and parents’ employers), and school sickness policies also impact the risk of presenteeism. To reduce presenteeism, parents, school staff and children need education about the impacts of attending school whilst unwell, clear guidance about the signs and symptoms of illness and organisational support.

## Electronic supplementary material

Below is the link to the electronic supplementary material.

Supplementary Material 1

Supplementary Material 2

## Data Availability

All data generated or analysed during this study are included in this published article and its supplementary information files.
