# Supplementary material for: Risk factors for school-based presenteeism in children: a systematic review
Source: BMC Psychol. 2023 May 23;11:169. doi: 10.1186/s40359-023-01207-1 (PMC10204673; doi:10.1186/s40359-023-01207-1)
Supplement: Supplementary file 1 — Supplementary Material 1 [file 40359_2023_1207_MOESM1_ESM.docx]

**Additional file 1: Search strategy**

**Medline**

1. Exp Nurseries/ or nurser*.mp.
2. School*.mp.
3. *school/
4. Exp child, preschool/ or exp child care/ or exp child day care centers/ or childcare.mp.
5. 1 or 2 or 3 or 4
6. Absenteeism.mp. or exp absenteeism/
7. Exp sick leave/ or presenteeism.mp. or exp presenteeism/
8. “sick leave”.mp. or exp sick leave/
9. Presenteeism.mp. or exp presenteeism/
10. Attendance.mp.
11. “school absence”.mp.
12. “authorised absence” .mp.
13. “authorized absence” .mp.
14. “unauthorised absence” .mp.
15. “unauthorized absence” .mp.
16. “nursery absence” .mp.
17. 6 or 7 or 8 or 9 or 10 or 11 or 12 or 13 or 14 or 15 or 16
18. 5 and 17

**PsychInfo**

1. Exp Nurseries/ or nurser*.mp.
2. School*.mp.
3. *school/
4. Exp child, preschool/ or exp child care/ or exp child day care centers/ or childcare.mp.
5. 1 or 2 or 3 or 4
6. Absenteeism.mp. or exp absenteeism/
7. Exp sick leave/ or presenteeism.mp. or exp presenteeism/
8. “sick leave”.mp. or exp sick leave/
9. Presenteeism.mp. or exp presenteeism/
10. Attendance.mp.
11. “school absence”.mp.
12. “authorised absence” .mp.
13. “authorized absence” .mp.
14. “unauthorised absence” .mp.
15. “unauthorized absence” .mp.
16. “nursery absence” .mp.
17. 6 or 7 or 8 or 9 or 10 or 11 or 12 or 13 or 14 or 15 or 16
18. 5 and 17

**Psyc Articles**

1. nurser*.mp.
2. School*.mp.
3. Pre-school .mp.
4. Child* .mp.
5. Secondary school.mp.
6. Primary school.mp.
7. 1 or 2 or 3 or 4 or 5 or 6
8. presenteeism.mp.
9. absenteeism.mp.
10. “sick leave”.mp.
11. “authorised absence” .mp.
12. “authorized absence” .mp.
13. “unauthorised absence” .mp.
14. “unauthorized absence” .mp.
15. “nursery absence” .mp.
16. 8 or 9 or 10 or 11 or 12 or 13 or 14 or 15
17. 7 and 16

**Child Development and adolescence development**

S1 TX nurser* OR TX school* OR TX preschool OR TX child*

S2 TX (absenteeism or attendance) OR TX presenteeism OR TX (sick leave or sickness absence) OR TX (school absenteeism or school absence) OR TX authorized absence OR TX unauthorized absence or TX nursery absence

S3 S1 AND S2

**Web of Science**

# 1 TS= (nurser* or school* or *school or child*)

# 2 TS= (absenteeism or presenteeism or "sick leave" or "school attendance" or "school absence" or "authorized absence" or "unauthorized absence" or "nursery absence")

# 3 #2 AND #1
