# Supplementary material for: Risk factors for school-based presenteeism in children: a systematic review
Source: BMC Psychol. 2023 May 23;11:169. doi: 10.1186/s40359-023-01207-1 (PMC10204673; doi:10.1186/s40359-023-01207-1)
Supplement: Supplementary file 2 — Supplementary Material 2 [file 40359_2023_1207_MOESM2_ESM.docx]

**Additional file 2: Data extraction table**

| Citation | Country | Study design (how school presenteeism was reported) | Sample characteristics (sample size; mean age and/or range; % female, % male) | Type of school setting (children characteristics) | Illness | Outcome(s) (reason or risk factors for school presenteeism) |
| --- | --- | --- | --- | --- | --- | --- |
| (Carroll et al., 2018) | UK | Discrete choice experiment (intentions about school presenteeism in a given scenario) | Parents (n = 122; 18-45 years; 96% female, 4% male) | Formal pre-school (children under four years; mean (range) age of child(ren) by number of children in household, 2.7 years; gender not reported) | A scenario describes a “marginally unwell” child. The child has a runny nose, slight cough, and high temperature, and no sickness or diarrhoea. Paracetamol/ibuprofen are given to the child to manage the temperature. | The probability of sending a marginally unwell child to pre-school was 43%.  Attributes that impacted a parents’ decision to send children to pre-school when unwell:  1) ability to swap session, number of sessions per year (0 = 40%; 5 = 45%; 10 = 45%; 20 = 41%)  2) quiet room (no quiet room = 36%; quiet room = 53%)  3) Paracetamol guidelines (paracetamol not allowed = 25%; paracetamol allowed = 62%)  4) fee reimbursement, number of sessions per year (0 = 49%; 5 = 44%; 10 = 39%; 20 = 40%) |
| (Carroll et al., 2016) | UK | Semi-structured interviews (previous decisions and intentions about school presenteeism) | Parents (n = 31; mean = 34 years; range = 26-47 years; 97% female, 3% male) | Formal pre-school (children aged between nine months and four years; mean 2.5 years; gender not reported) | Sending children to pre-school with respiratory tract infections (RTIs) | Themes identified that impacted parents’ decision to send children to pre-school:  1) Lay perceptions of RTIs (decisions about gastrointestinal symptoms appeared much easier to make than decisions about respiratory symptoms; parents more likely to send children to school with RTIs, due to: belief that colds are highly prevalent, caught from anywhere and not preventable; adults with colds still expected to attend work; individual can still carry out normal activities with a cold; a cold cannot be treated so should not affect daily life) 2) Other parents' decisions (participants recognised that other parents would face the same difficulties as them re: decisions about exclusion and so tried not to judge others whilst another parent said they would be annoyed if their child caught something from another child who should have been kept home) 3) Nursery sickness/exclusion policies (policies reported to be vaguer when it comes to respiratory symptoms/conditions than gastrointestinal; confusion about some of the policies and what conditions/symptoms would be subject to regulation or control; parents reported a sense that respiratory illnesses are not of concern to nurseries)  4) Practical considerations and pressures to send children to nursery (work issues; financial penalties; availability of alternative care) 5) Potential nursery policy changes (discount in fees for absences, or being able to take other sessions on different days instead, would make people happier to keep their kids home) |
| (Copeland, Duggan, & Shope, 2005) | US | Cross-sectional survey (intentions about school presenteeism for a specific symptom of illness) | Parents (n = 142; age not reported; 91% female, male not reported)  School staff (n = 36; 49% female, male not reported) | Formal pre-school (ages unclear, alludes to children under 5 years old; gender not reported). | Sending children to pre-school using a list of 12 common symptoms that require exclusion (9) or that do not warrant exclusion (3) derived from the American Academy of Paediatrics (AAP)/ the American Public Health Association (APHA) guidelines. | Knowledge about exclusion processes (mean +/- standard deviation):  Parents = 64.1 +/- 13.1  Pre-school staff = 63.4 +/- 12.8  Symptoms that require exclusion from pre-school and the percentage of participants that indicated exclusion:  1) signs of illness that prevent participation in normal activity (parents = 74%; pre-school staff = 72%)  2) an illness that requires greater care than pre-school staff can provide (parents = 95%; pre-school staff = 94%)  3) oral temp ≥ 101℉ (parents = 94%; pre-school staff = 99%)  4) axillary temp ≥ 100℉ (parents = 82%; pre-school staff = 85%)  5) uncontrolled coughing (parents = 72%; pre-school staff = 65%)  6) persistent crying (parents = 50%; pre-school staff = 37%)  7) Wheezing (parents = 68%; pre-school staff = 75%)  8) more than 3 loose stools in 24 hours (parents = 76%; pre-school staff = 89%)  9) two or more vomiting episodes in 24 hours (parents = 88%; pre-school staff = 93%)  Symptoms that participants (%) would exclude from pre-school that do not require exclusion:  1) new rash without fever or behaviour change (parents = 66%; pre-school staff = 69%)  2) thick green or yellow discharge from nose for 5 days (parents = 78%; pre-school staff = 84%)  3) redness of eyes and watery eye discharge (parents = 88%; pre-school staff 91%)  Beliefs about exclusion:  1) Sick children need more care and attention than CCPs can give (parents = 85; pre-school staff = 92)  2) Sick children spread disease to other children (parents = 91; pre-school staff = 96)  3) Sick children spread disease to pre-school staff (parents = 86; pre-school staff = 97)  4) Sick children need to be at home to recover faster (parents = 72; pre-school staff = 87)  5) Sick children need to be at home for their own comfort (parents = 80; pre-school staff = 87)  6) Pre-schools may be held legally responsible if something bad happens to a sick child (parents 60; pre-school staff = 70)  7) Pre-schools do not have enough knowledge to care for mildly ill children (parents 27; pre-school staff = 59)  Other statements:  1) Pre-school staff use good judgement regarding which children need to be excluded (parents = 68%: pre-school staff = 87%)  2) Pre-school staff follow the written exclusion guidelines closely (parents = 78%; pre-school staff = 86%)  3) Pre-school staff are consistent about which sick kids need to be excluded and which do not (parents = 68%; pre-school staff = 85%)  4) Current day care policies are too vague (parents = 31%; pre-school staff = 51%)  5) In general, employers support the need for parents to care for their sick child at home (parents = 76%; pre-school staff = 60%)  6) The way the day-care handles ill children negatively affects parent’s (my) job success or career advancement (parents = 17%; pre-school staff = 28%) |
| (Copeland, Harris, Wang, & Cheng, 2006) | US | Cross-sectional survey (intentions about school presenteeism for a given scenario) | Parents (n = 223; age not reported; 92% female, male not reported)  School staff (n = 192; age and gender not reported; 98% female, male not reported) | Formal pre-school (children aged under five years; gender not reported). | Vignettes describing six common childcare illness:  upper respiratory infection (runny nose, dry cough and temperature); conjunctivitis (red eyes, eye drainage and temperature); gastroenteritis (vomiting, two loose stools, temperature); mild febrile illness (temperature, sleepy and unwilling to play with toys); tinea capitis (circular area on scalp, little red bumps and flakes); atopic dermatitis (red bumpy rash over thighs and back of the hands, dry skin). | Parents (61%) and pre-school staff (60%) had similar rates of adherence to pre-school exclusion guidelines.  Percentage that participants were adherent to the exclusion guidelines for each illness:  1) upper respiratory infection (parents = 63%; pre-school staff = 62%)  2) conjunctivitis (parents = 67%; pre-school staff = 75%)  3) gastroenteritis (parents = 69%; pre-school staff = 69%)  4) mild febrile illness (parents =74; pre-school staff = 69)  5) tinea capitis (parents = 37%; pre-school staff = 21%)  6) atopic dermatitis (parents = 60%; pre-school staff = 66%)  Factors that impacted adherence:  1) temperature presented (no temperature; ˂ 100℉; ˃ 101.5℉) impacted adherence. Adherence varied by participant type and illness.  2) symptom severity (less-severe; more-severe symptoms) impacted adherence. Adherence varied by participant type and illness. |
| (Friedman, Lee, Kleinman, & Finkelstein, 2003) | US | Cross-sectional survey (intentions about school presenteeism for a given scenario) | Parents (n = 211; age not reported; 96% female, male not reported)  School staff (n = 85; age and gender not reported) | Formal pre-school (children aged between six and 48 months) | Upper respiratory infections and three specific symptoms (clear runny nose, green runny nose and cough without difficulty breathing) without a high temperature. | Symptoms that indicated exclusion from pre-school:  1) clear runny nose (parents = 5%; pre-school staff = 0%)  2) green runny nose (parents = 35%; pre-school staff = 28%)  3) cough without difficulty breathing (parents = 15%; pre-school staff 35%)  *(Some %s are approximate as they are not stated in the text and are inferred from a graph so may not be exact)* |
| (Johansen, 2015) | Norway | Cross-sectional survey (previous school presenteeism) | Children lower secondary school (LSS) (n = 1841; range = 15-16 years; female not reported, 52% male)  Children upper secondary school (USS) (n = 1148, range = 17-18 years; female not reported; 50% male) | Secondary school | Non-specified illness (participants were asked how many times, if any, in the last school year, they had attended school “despite feeling so ill that they should have taken sick leave”) | Reported school presenteeism (%) per episode by children’s age:  0 episodes (LSS = 25%; USS = 20%)  1-3 episodes (LSS = 51%; USS = 47%)  ≥ 4 episodes (LSS = 24%; USS = 33%)  Factors that had a significant (*p* < 0.01 or *p* < 0.05*) impact on ≥ 4 episodes of school presenteeism (adjusted odd ratio, 95% CI):  1) female compared to male: LSS = 1.45 (1.17 to 1.95); USS = 1.45 (1.10 to 1.91)  2) high school absence compared to no/low school absence: LSS = 1.66 (1.25 to 2.19); USS = 1.96 (1.47 to 2.60)  3) school motivation (scale: 1 = high motivation, 5 = low motivation): LSS = 0.80 (0.69 to 0.92)  4) vocational studies compared to general studies: USS = 1.36* (1.00 to 1.85) |
| (Johansen, 2018) | Belgium; Estonia; Finland; Italy; Latvia | Cross-sectional survey (previous school presenteeism) | Children (n = 2417; mean 16.9 years; range = 16-19 years; 49% female, male not reported) | Secondary school | Non-specified illness (participants response to “during the last school year, did you go to school despite feeling so ill that you should have stayed home sick?”). | Two or more episodes of school presenteeism in the previous school year was reported in 48% of children.  Identified two types of motivations for engaging in school presenteeism  1) intrinsic motivations (IM) = well-being at school and engaged in school presenteeism because it was personally rewarding  2) extrinsic motivations (EM) = attendance pressure and engaged in school presenteeism because of external rewards  Motivations for school presenteeism (IM or EM, %):  1) crucial materials/syllabus is explained at school (EM, 68%)  2) high school absence might negatively affect grades (EM, 67%)  3) school attendance requirements (EM, 50%)  4) maintain social network (IM, 41%)  5) do not want to burden classmates (not included in results, 40%)  6) high school absence might negatively affect chances to get a job/apprenticeship (EM, 40%)  7) great interest in what is learned at school (IM, 40%)  8) enjoy going to school (IM, 26%)  9) pride depends on not being sick from school (IM, 22%)  10) school is beneficial to health (IM, 20%)  11) other reasons (not included in the results, 14%)  Factors that had a significant (*p* < 0.01) higher score on intrinsic motivation for presenteeism (unstandardised coefficients, 95% CI):  1) females compared males: -0.09 (-0.16 to 0.02)  2) vocational compared to academic/technical: 0.23 (0.15 to 0.31)  3) immigrants compared to non-immigrant: 0.20 (0.09 to 0.31)  4) highly educated parents compared to low educated parents: -0.10 (-0.17 to 0.04)  Factors that had a significant (*p* < 0.01) higher score on extrinsic motivation for presenteeism (unstandardised coefficients, 95% CI):  1) females compared to males: 0.09 (0.04 to 0.14)  2) high school absence compared to no/low school absence: 0.10 (0.04 to 0.16) |
| (Johansen, 2019) | Belgium; Estonia; Finland; Italy; Latvia | Repeated measures survey (previous school presenteeism) | Children time 1, year 2016 (T1) (n = 7008; range 16-19 years; gender not reported)  Children time 2 year, 2017 (T2) (n = 5002; range 16-19 years; gender not reported) | Secondary school | Non-specified illness (participants were asked how many times, if any, in the last school year, they had attended school “despite feeling so ill that they should have taken sick leave”) | Reported school presenteeism (%) of children per episode by time:  0 episodes (T1 = 21%; T2 = 20%)  1-4 episodes (T1 = 63%; T2 = 65%)  ≥ 5 episodes (T1 16%; T2 = 15%)  Factors that had a significant (*p* < 0.01 or *p* < 0.05*) impact on high school presenteeism which was considered ≥ five episodes (adjusted odd ratio, 95% CI):  1) age, youngest to oldest: T1 = 1.09* (1.02 to 1.19); T2 = 1.12* (1.02 to 1.23)  2) immigrant compared to native: T2 = 1.33* (1.01 to 1.76)  3) high school sickness absence compared to no/low school sickness absence: T1 = 1.86 (1.61 to 2.14); T2 = 2.12 (1.77 to 2.55)  4) school motivation (scale: 1 = low motivation, 5 = high motivation): T1 = 1.16 (1.07 to 1.26); T2 = 1.18 (1.05 to 1.33)  5) Estonia compared to Finland: T1 = 2.19 (1.69 to 2.85); T2 = 1.53 (1.14 to 2.06)  6) Italy compared to Finland: T1 = 1.58 (1.25 to 2.02); T2 = 1.59 (1.17 to 2.56)  7) Latvia compared to Finland: T1 = 3.45 (2.79 to 4.27); T2 = 2.65 (2.01 to 3.50) |
| (King & Leask, 2018) | Australia | Semi-structured interviews (previous decisions and intentions about school presenteeism) | Parents (n = 42; exact ages not reported, lowest age was “under 21” and oldest age was 40-50 years; female 98%, 2% male) | Formal pre-school (children under five years; gender not reported) | Infectious illness and disease prevention (participants were asked when they think their child was “too sick” to attend childcare) | Themes that impacted when parents perceived children too ill to attend pre-school:  1) 'Vitamin dirt' - natural agents and health (need for positive germ exposure to build immune system) 2) Contagion (views on disease transmission; gastrointestinal symptoms seen as more serious than colds or influenza) 3) Preventive health behaviours (separating child from others was done when parents believed their child was contagious; unspoken social contract where they believed other parents would reciprocate) 4) Interaction of beliefs with childcare attendance decisions (judgement to exclude or not was based on parental instinct; none mentioned official childcare guidelines; some mention of difficulties for working parents) |
| (Landis, Earp, & Sharp, 1988) | US | Cross-sectional survey (intentions about school presenteeism for specific symptoms) | Parents (n = 134; median age = 32 years; 100% female, males not included in the study)  School staff (n = 302; median age = 28 years; 99% female, males not reported) | Formal pre-school (children aged between two and five years) | Common childhood infections (participants were asked when a child should be excluded from childcare when presented with eight symptoms, a new runny nose, new cough, unusually cranky, ear pain, sore throat, skin rash, diarrhoea and conjunctivitis and four different temperature ranges that often occur with infections) | Factors that impacted when participants would request “immediate pickup” from pre-school due to illness (%):  1) child has a high temperature:  i) 99℉-99.9℉ (parents = 3%; SS = 11%)  ii)100℉-100.9℉ (parents = 26%; SS = 41%)  iii)101℉-101.9℉ (parents = 71%; SS = 74%)  iv) ≥ 102℉ (parents = 93%; SS = 97%)  2) specific symptoms when child does not have a fever:  i) conjunctivitis (parents = 46%; pre-school staff = 64%)  ii) diarrhoea (parents = 27%; pre-school staff = 61%)  iii) skin rash (parents = 21%; pre-school staff = 22%)  iv) ear pain (parents = 12%; pre-school staff = 18%)  v) sore throat (parents = 8%; pre-school staff = 18%)  vi) unusually cranky (parents = 2%; pre-school staff = 3%)  vii) new cough (parents = 1%; pre-school staff = 2%)  viii) runny nose (parents = 1%; pre-school staff = 1%)  3) specific symptoms when child has a temperature of 101℉-101.9℉:  i) conjunctivitis (parents = 57%; pre-school staff = 79%)  ii) diarrhoea (parents = 57%; pre-school staff = 77%)  iii) skin rash (parents = 45%; pre-school staff = 61%)  iv) ear pain (parents = 52%; pre-school staff = 66%)  v) sore throat (parents = 45%; pre-school staff = 65%)  vi) unusually cranky (parents = 32%; pre-school staff = 44%)  vii) new cough (parents = 28%; pre-school staff = 45%)  viii) runny nose (parents = 27%; pre-school staff = 44%) |
| (Levy, Murphy, Kamp, Langer, & van Tilburg, 2021) | US | Cross-sectional survey (previous school presenteeism) | Parents (n = 227; 135 were mothers of only children and 92 had multiple children; mean age 44.35 for mothers of only children and 42.55 for mothers of multiple children; 100% female, males not included) | School not specified (Children aged between eight and fifteen years; mean = 12.03 years; female not reported, 48.8% male) | Only children compared to children with siblings with stomach-ache or abdominal pain in the past three months (participants were asked about their response to their child’s symptoms) | Parents were scored on how much they encouraged (protectiveness score) and criticised (minimisation score) child illness behaviour.  Protectiveness = parents of only children scored (mean) lower on the protectiveness scale compared to parents of children with siblings (1.71 versus 1.78, *p* = 0.420)  Minimisation = parents of only children scored higher on the minimisation scale compared to parents of children with siblings (1.13 versus 0.98, *p* = 0.055)  1) Only children were less likely to miss school compared to children with siblings (19.0% versus 33.2%, *p* < 0.01) due to stomach-ache or abdominal pain. |
| (Polyzoi, Babb, & Babb, 2003)* | Canada | Mixed methods: cross-sectional survey and interviews (previous experience and intentions about school presenteeism) | Parents (n = 742; age and gender not reported)  School staff (n = 328; age and gender not reported) | Formal and informal pre-school (children characteristics were not reported) | Caring for “mildly ill” children (e.g., cold, influenza, ear infections, chicken pox and conjunctivitis) among working parents. | Challenges that working parents face when their children are too ill for pre-school:  1) costs associated with staying home to care for their ill child  2) fear of job loss  3) worry about the well-being of their child, if they go to work  Concerns to pre-school staff about providing care for mildly ill children:  1) parental pressure to accommodate ill children,  2) risk of cross-infection from ill children  3) increased caregiver workload and lack of space and resources  4) Liability and lack of legislation and funding to support the care of ill children. |
| (Prout, 1986) | UK | Ethnography case-study (previous experience and intentions about school presenteeism) | One primary school class in year 4 (n = 35, children; n = 1 teacher; presumably between 10 and 11 years old, mean child ages = 11 years; “almost equally divided by boys and girls”) | Primary school | Excluding children from primary school when they “claim they are sick” (observations about the children and school staff perspective). | Every case of school sickness absence in a five-month period was assessed (using observation, interviews, health diaries, and school documents).  Theme of indulgent mothers and *wet* children were identified regarding observations from teachers and school staff in response to ill children:  1) Most mothers were thought of by school staff as too ready to keep their children off school; theme of 'maternal over-anxiety' - especially in working class mothers.  2) School staff suggested children's illnesses could be caused and prolonged by mothers' anxiety.  3) children’s claims about illness were often rebuffed, particularly if they were perceived as wet by school staff; unless the child was dramatic and threatening and symptoms were visible.  4) Children had to make at least 3 approaches to their teacher to claim sickness; they were first told to simply 'wait and see what happens'. Boys were more likely to be believed as 'they don't moan about things'. Older boys more likely to be believed than younger boys; seen as 'sensible'.  5) a child’s perceived wetness by school staff was based on the children’s age (young children were more wet than older ones) and gender (girls were more wet than boys).  6) Child's demeanour could have an impact - e.g., one girl was not believed to be ill because she was 'giggling'.  7) Children seen as 'well until proven ill'. It was expected that most would try to feign illness at some point. |
| (Prout, 1988) | UK | Ethnography case-study (previous experience and intentions about school presenteeism) | One primary school class (n = 35, children; n = 1 teacher; presumably between 10 and 11 years old, mean child ages = 11 years; “almost equally divided by boys and girls”) | Primary school | Mothers’ decision to keep children “off school sick” (observations about the mother’s perspective). | Every case of school sickness absence in a five-month period was assessed (using observation, interviews, health diaries, and school documents).  Themes that were identified from observations and interviews with mothers about school presenteeism:  1) Illness and the detection of feigning (parents’ main assumption was that children were feign symptoms of illness and first stage was to decide if their children could be believed; parents suggest they could tell illness in their children but not other children; the visibility of symptoms indicated illness)  2) emotional upset and training in stoicism (parents tried to identify weather the illness was due to an emotional disturbance; parents suggested that children were too ready to “give in” to feelings of unwell and they would encourage their children to be “strong” and “snap out of it”)  3) childhood career and the meaning of sickness (the transition from primary school to secondary school was considered moving from childhood to adulthood and parents and school staff, which increased the options about encouraging stoical attitudes)  4) child illness as deviance (child illness relates to parental competence, having “normal healthy children” suggested a good mother and the admission of too many symptoms impacts that perception.  5) schools, mothers and sickness absence (parents have a parental responsibility to ensure their children attend school; parents felt they may be perceived as incompetent or neglectful if their children were perceived as “sickly” by school staff)  6) the child as social actor: a reprise (children were denied any formal power in the decision making process; children reported successful instances of feigning illness by manipulating signs and symptoms of illness that were both realistic but not too threatening) |
| (Rooshenas, Wood, Brookes-Howell, Evans, & Butler, 2014) | UK | Mixed methods: cross-sectional survey, and interviews (previous experience and intentions about school presenteeism) | Parents, interview only (n = 29; mean 36 years; range 23-46 years)  Formal school staff (n = 77, survey; n = 15, interviews; age and gender not reported)  Informal school staff (n = 140 survey; n = 9, interviews; age and gender not reported) | Formal and informal pre-school (children aged five and under) | Children who had been excluded from pre-school due to an infectious illness (e.g., whooping cough, parasitic infections, and scarlet fever). | Reasons for school presenteeism (theme identified and %, when reported):  1) policy content did not mention specific infections and criteria for exclusion and/or readmittance (18% formal school; 41% = informal school)  2) parents and school staff indicated that written policies were an accurate reflection of their data-to-day practices  3) School staff’s verbal advice to parents impacted parents’ response to children’s illness.  4) type of symptoms impacted parents’ and school staff’s response to children’s illness  5) parents’ experiences of exclusion and/or readmittance (parents reported seeking antibiotic treatment solely to prevent exclusion/or expediting their child’s readmittance to pre-school) |
| (Skull, Ford-Jones, Kulin, Einarson, & Wang, 2000) | Canada | Cross-sectional survey (previous experiences and intentions about school presenteeism) | School staff (n = 36; age and gender not reported) | Formal pre-school (children in diapers (i.e., nappies), age not reported) | Upper respiratory infections (URI) (participants were asked reasons for exclusion in most recent case, and seven symptoms, ear pain, green or yellow nasal discharge, cough with phlegm, unusual behaviour, sore throat, runny nose and dry cough). | Most (69%) pre-schools had a written policy about managing children with URIs.  Factors that school staff reported would usually or always result in exclusion for diapered children with URIs:  1) Symptoms:  i) ear pain (64%)  ii) green or yellow nasal discharge (56%)  iii) cough with phlegm (44%)  iv) unusual behaviour (42%)  v) sore throat (31%)  vi) runny nose (19%)  vii) dry cough (14%)  2) if the child was unable to participate in activities (92%)  3) to prevent the spread of infection (67%)  4) insufficient staff to provide adequate care (61%)  Factors that SSs reported had prevented them from excluding diapered children with URIs:  1) child had a prescription for antibiotics (69%)  2) parents did not want their child excluded (64%)  3) parent could not stay home from work (14%) |
| (Sticher, Bielicki, & Berger, 2018) | Switzerland | Cross-sectional survey (previous experiences about school presenteeism) | School staff (n = 249, managing a total of 6,424 children; age and gender not reported) | Formal pre-school (children aged five years and under) | Ill children (participants were asked how they make the decision to exclude a child who is “ill” and how they manage parents who do not accept the decision to exclude). | Most (85%) schools had a standard policy for managing ill children.  Exclusion criteria reported by school staff – medical and social circumstances (%):  1) illness perceived as contagious (52.2%)  2) illness severity (45.3%)  3) institutional limits (8.4%)  4) interference with other children’s needs (3.2%)  Exclusion criteria reported by school staff – symptoms and signs (%):  1) fever (87.4%)  2) conjunctivitis (29.3%)  3) vomiting (19.7%)  4) diarrhoea (18.0%)  5) other symptoms (abnormal breathing and rash) (9.6%)  Ambiguities in school staff’s decision making about excluding ill children from pre-school:  1) drugs that prevent fever (23.0%)  2) whether certain symptoms were related to teething (5.2%)  3) difficulties or insufficient communication with parents (13.6%)  4) lack of medical knowledge (12.6%)  5) parents are unable to find alternative care (5.2%) |
| (Thomson, Henderson, & Smith-Palmer, 2019) | UK | Mixed methods: epidemiological data and interviews (previous experience of school presenteeism) | Children (mean = 35 months; range 18 to 58 months; gender not reported)  Pre-school with 92 children and 19 staff members, n = 14 children, and n = 1 adult (family member) with confirmed Salmonella | Formal pre-school (children aged five years and under) | Salmonella infection confirmed via stool samples and gastrointestinal infection symptoms (GI) (nausea, vomiting, diarrhoea, and/or abdominal pain). | School presenteeism (attended pre-school either with GI or within 48 hours following recovery of GI) was reported in 50% (n = 7) of confirmed child cases.  Factors impacting school presenteeism and Salmonella outbreak:  1) symptom severity - minority of confirmed child cases (n = 5, 35.7%) were excluded by day-care staff after identifying loose stools. Parents did not report GI to pre-school staff because the symptoms were not severe enough – although they were aware of the ongoing Salmonella outbreak  2) alternative childcare - parents commonly reported the motivating factor for sending children to pre-school whilst unwell was because of difficulty in finding alternative childcare and / or unable to take leave from employment. |

* Data was originally discussed in an earlier report but a full-text was not available

**References**

Carroll, F. E., Al-Janabi, H., Rooshenas, L., Owen-Smith, A., Hollinghurst, S., & Hay, A. D. (2018). Parents' preferences for nursery care when children are unwell: a discrete choice experiment. *Journal of Public Health, 20*, 20. doi:<https://dx.doi.org/10.1093/pubmed/fdy215>

Carroll, F. E., Rooshenas, L., Owen-Smith, A., Al-Janabi, H., Hollinghurst, S., & Hay, A. D. (2016). Factors influencing parents' decision-making when sending children with respiratory tract infections to nursery. *Journal of Public Health, 38*(2), 281-288. doi:<https://dx.doi.org/10.1093/pubmed/fdv037>

Copeland, K. A., Duggan, A. K., & Shope, T. R. (2005). Knowledge and Beliefs About Guidelines for Exclusion of Ill Children From Child Care. In (Vol. 5, pp. 365-371).

Copeland, K. A., Harris, E. N., Wang, N.-Y., & Cheng, T. L. (2006). Compliance With American Academy of Pediatrics and American Public Health Association Illness Exclusion Guidelines for Child Care Centers in Maryland: Who Follows Them and When? *PEDIATRICS, 118*(5), e1369-e1380. doi:10.1542/peds.2005-2345

Friedman, J. F., Lee, G. M., Kleinman, K. P., & Finkelstein, J. A. (2003). Acute Care and Antibiotic Seeking for Upper Respiratory Tract Infections for Children in Day Care. *Archives of Pediatrics & Adolescent Medicine, 157*(4), 369. doi:10.1001/archpedi.157.4.369

Johansen, V. (2015). Sick and still at school: an empirical study of sickness presence among students in Norwegian secondary school. *BMJ Open, 5*(9), e008290. doi:<https://dx.doi.org/10.1136/bmjopen-2015-008290>

Johansen, V. (2018). Motives for sickness presence among students at secondary school: a cross-sectional study in five European countries. *BMJ Open, 8*(1), e019337. doi:<https://dx.doi.org/10.1136/bmjopen-2017-019337>

Johansen, V. (2019). A PANEL STUDY OF SICKNESS PRESENCE AND SICKNESS ABSENCE AMONG SECONDARY SCHOOL STUDENTS. *International Journal of Occupational Medicine and Environmental Health, 32*(6), 797-804. doi:10.13075/ijomeh.1896.01441

King, C. L., & Leask, J. (2018). Parental disease prevention health beliefs and triggers for keeping children home from childcare-a qualitative study in Sydney, Australia. *Child: Care, Health & Development, 44*(2), 326-331. doi:<https://dx.doi.org/10.1111/cch.12481>

Landis, S. E., Earp, J. A. L., & Sharp, M. (1988). Day-Care Center Exclusion of Sick Children: Comparison of Opinions of Day-Care Staff, Working Mothers, and Pediatricians. *PEDIATRICS, 81*(5), 662-667. Retrieved from <https://pediatrics.aappublications.org/content/81/5/662.long>

Levy, R. L., Murphy, T. B., Kamp, K., Langer, S. L., & van Tilburg, M. A. L. (2021). Parental Response to Only Children: Breaking the Stereotypes. *Children, 8*(7), 17. doi:<https://dx.doi.org/10.3390/children8070605>

Polyzoi, E., Babb, J.-A. V., & Babb, J. C. (2003). The Care of Mildly Ill Children: Perspectives of Canadian Parents and Childcare Directors. *Journal of Early Childhood Research, 1*(2), 213-225. doi:10.1177/1476718X030012005; 15 10.1177/1476718X030012005

Prout, A. (1986). ‘Wet children’ and ‘little actresses': going sick in primary school. *Sociology of Health & Illness, 8*(2), 113-136. doi:10.1111/j.1467-9566.1986.tb00028.x

Prout, A. (1988). Off School Sick: Mothers' accounts of School Sickness Absence. *Sociological review, 36*, 765-789.

Rooshenas, L., Wood, F., Brookes-Howell, L., Evans, M. R., & Butler, C. C. (2014). The influence of children’s day care on antibiotic seeking: a mixed methods study. *British Journal of General Practice, 64*(622), e302-e312. doi:10.3399/bjgp14x679741

Skull, S. A., Ford-Jones, E. L., Kulin, N. A., Einarson, T. R., & Wang, E. E. L. (2000). Child Care Center Staff Contribute to Physician Visits and Pressure for Antibiotic Prescription. *JAMA Pediatrics, 154*(2), 180-183. doi:10.1001/archpedi.154.2.180

Sticher, B., Bielicki, J., & Berger, C. (2018). Temporary exclusion of ill children from childcare centres in Switzerland: practice, problems and potential solutions. *BMC Health Services Research, 18*(1), 25. doi:<https://dx.doi.org/10.1186/s12913-018-2831-5>

Thomson, R. M., Henderson, H. J., & Smith-Palmer, A. (2019). An outbreak of Salmonella Saintpaul in a Scottish childcare facility: the influence of parental under-reporting. *BMC Infectious Diseases, 19*(1), 847. doi:<https://dx.doi.org/10.1186/s12879-019-4516-z>
